# Estimate of Coffin–Manson Curve Shift for the Porous Alloy AlSi9Cu3 Based on Numerical Simulations of a Porous Material Carried Out by Using the Taguchi Array

**DOI:** 10.3390/ma15062269

**Published:** 2022-03-18

**Authors:** Dejan Tomažinčič, Jernej Klemenc

**Affiliations:** Faculty of Mechanical Engineering, University of Ljubljana, Aškerčeva 6, SI-1000 Ljubljana, Slovenia; jernej.klemenc@fs.uni-lj.si

**Keywords:** low-cycle fatigue, aluminium alloy, porosity, Coffin–Manson relationship, Taguchi, multivariate regression analysis

## Abstract

In real engineering applications, machine parts are rarely completely homogeneous; in most cases, there are at least some minor notch effects or even more extensive inhomogeneities, which cause critical local stress concentrations from which fatigue fractures develop. In the present research, a shift of the Coffin–Manson *ε*_a_–*N* material curve in a structure with random porosity subjected to dynamic LCF loads was studied. This allows the rest of the fatigue life prediction process to remain the same as if it were a homogeneous material. Apart from the cyclic *σ*–*ε* curve, which is relatively easy to obtain experimentally, the *ε*_a_–*N* curve is the second most important curve to describe the correlation between the fatigue life *N* and the strain level *ε*_a_. Therefore, the correct shift of the *ε*_a_–*N* curve of the homogeneous material to a position corresponding to the porous state of the material is crucial. We have found that the curve shift can be efficiently performed on the basis of numerical simulations of a combination of five porosity-specific geometric influences and the associated regression analysis. To model the modified synthetic *ε*_a_–*N* curve, five geometric influences of porosity by X-ray or *μ*-CT analysis are quantified, and then the porosity-adjusted coefficients of the Coffin–Manson equation are calculated. The proposed approach has been successfully applied to standard specimens with different porosity topography.

## 1. Introduction

Porosity is a constant issue in the foundry industry [1,2]. Although some progress has been made in estimating the durability of porous structures [3,4,5], new approaches are still being sought to improve fatigue life prediction further. The larger scatter of test results and consequently, larger discrepancies in predictions are mainly caused by the randomness of the porosity phenomenon [6], which is difficult to evaluate in large-scale production of castings, because the porosity is not always of the same size and not always in the same position within the load-bearing structure. By using the established continuum damage mechanics (CDM) [7,8,9], porosity can be addressed in two ways. Porosity can be accurately reconstructed after *μ*-CT examination, and stress analysis can be performed on digital twins of porous structures [10,11]. The increased stresses *σ* at the pores are then transformed to equivalent strain levels *ε*_a_ using the *σ*–*ε* cyclic curve and finally transformed to the number of cycles *N* by using the *ε*_a_–*N* curve of the homogeneous material. This procedure is known as Method A in the literature [12,13,14]. Good predictions of the fatigue life can be expected due to the quality of the stress analysis, which is important in the damage calculation. However, there is a significant problem linked to the application of the CDM–Method A approach in practice. To use it the actual inhomogeneities actually need to be modelled, which was shown in two of our previous articles [11,14]. In our case, the corresponding finite element models for the clustered macro porosity consisted of approximately 900,000 finite elements and it took a long time to predict the fatigue life for a geometry that is very simple in practice (a cylinder). It was for this reason that our idea of predicting the fatigue life for homogenized structures with adapted fatigue-life curves was developed. Such a procedure is not outdated, because it is daily applied for the welded structures—see, for example, EUROCODE 3: Part 1–9 standards for steel. It is also embedded into every up-to-date commercial finite element code, which is used for fatigue-life calculation (e.g., Magna FEMFAT). In our opinion, CDM–Method A or X-FEM approaches have relatively low usage potential for the real-life structures (due to their complexity and longer processing times), which are often far more complex than simple or component-like specimens. Besides, it is very difficult to assure convergence of the X-FEM method for cyclic loads and structures with a real geometry. Lastly, X-FEM is based on the linear-elastic fracture mechanics principle, which validity is highly questionable in our case. Therefore, an alternative approach, named Method B in the literature [14], is increasingly being used. In this case, porosity is not taken into account directly by increased stresses around the pores as a consequence of notch effects but is instead included in the calculations by an appropriately reduced level of the *ε*_a_–*N* curve for the porous material. The main objective of the research is to adapt the fatigue-life curve of the porous material according to the actual porosity geometries. The main scientific contribution of this research is to prove that this is possible with a separate family of well-planned numerical simulations, which summarise the most important porosity parameters in a meaningful manner. On the basis of this simulation, a multivariate regressive model is set up that enables the calculation of the porosity-adapted fatigue-life curves.

After the introduction, the article consists of the presentation of the AlSi9Cu3 alloy material data and the experimental data on the strength of the analysed porous samples. Section 3 presents the derivation of the proposed regression model for the estimation of the shift of the Coffin–Manson curve of the porous material, followed by the analyses carried out and the presentation of the experimental/theoretical agreement of the results in Section 4. The paper ends with conclusions, acknowledgements and a list of references.

## 2. Materials and Methods

### 2.1. Methodology

**Figure 1:**
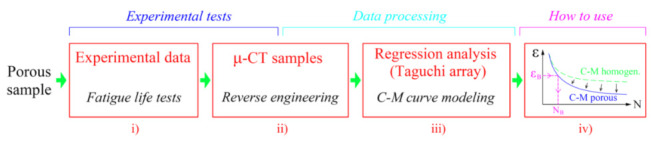
Flowchart of a procedure for estimation the fatigue life of a porous structure.

In the introductory part of the research, a methodology for predicting the service life in randomly porous alloys was first set up. The methodology is graphically shown in Figure 1. It is divided into four successive phases:

(i) From the experimental tests, the basic Coffin–Manson endurance curve of a homogeneous material is determined. The least-squares method is used to connect the experimental points into a uniform curve.
(ii) To analyse the porosity in the alloy, the geometry of the pores is determined by using the *μ*-CT examination and reverse engineering based on the voxel-processing technique.
(iii) This is followed by the most important phase, i.e., modelling the porosity of the adjusted C–M curve. Since the influence of notch effects can be estimated using FE simulations, a Taguchi matrix of randomly distributed pores was defined, which enabled the formation of the combinations of pore size, position, pore flatness, pore distance and pore orientation. These are the main effects that reduce the fatigue life of the porous alloy. Based on the FE simulations for every combination of pore geometry, the relationship between the five geometric influences and the parameters of the fatigue-life curve for the porous material are determined using a multivariate regression model. It shows the sensitivity of the fatigue life to the variation of the individual geometric parameters of the pores. Once all the regression model is built and the porosity state for a certain specimen/component is determined, the porosity-adjusted Coffin–Manson fatigue life curve can be modelled.
(iv) In the final stage, the prediction of the service life is based on the case-wise adjusted Coffin–Manson curve of the porous material. Based on this curve, the fatigue life of the *N*_B_ porous alloy can be estimated for a known load amplitude *ε*_B_.

The presented method is validated by comparing the predicted fatigue life to the experimental data for selected porous samples.

### 2.2. Application of Homogenised Approach

**Figure 2:**
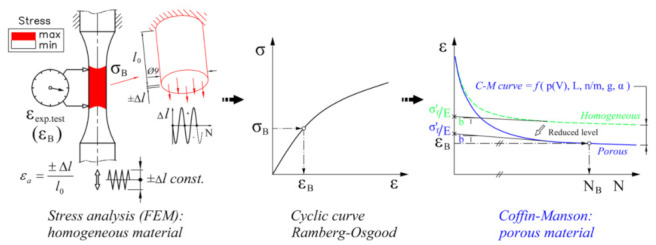
Sequential steps in predicting the fatigue life *N* of a porous structure using Method B.

The upgrade of the established procedure for the estimation of the fatigue life in Method B should result in a highly suitable tool for engineering applications in an industrial environment. The procedure, schematically shown in Figure 2, is based on assuming a theoretically homogeneous structure to predict fatigue life, while the influence of porosity is taken into account via a shift of the Coffin–Manson (hereinafter C–M) *ε*_a_–*N* curve of the material. The shift of the C–M curve is done due to the influence of various notch effects on the dynamic durability. There are several different approaches when dealing with notch effects [15,16,17] and estimating durability curves, taking crack growth into account [18]. We used the critical plane approach [19] of a dynamically loaded sample. The advantage of Method B is that after the C–M curve level has been lowered, a conventional procedure can be used to estimate the fatigue life of a porous structure by calculating the extent of damage achieved based on a simulation of the stabilised load cycle. This is carried out on the basis of a modified C–M curve, which is entered into software for fatigue life assessment, which works on the same basis; examples of such software include Siemens LMS Virtual.lab, Magna FEMFAT, Dassault Systèmes SIMULIA fe-safe, Ansys nCode DesignLife, etc. The main factor for the quality of the prediction is the correct shift of the *ε*_a_–*N* C–M curve of the homogeneous material to a lower level adapted to the state of porosity. Several procedures based on the concept of a synthetic curve shift have been proposed in the past [20], but they have been tested for steel alloys and applied to *S*–*N* curves (HCF region). Usually, they also require a series of additional experimental tests. In the design of the procedure for lowering the level of the durability curve, our objective was to use easily obtainable data from the analysed porous samples, so the necessary geometrical data on pore sizes, their position and orientation can be obtained by available non-destructive methods, such as X-ray imaging or by more advanced and accurate CT scans [21]. It should be noted that in our studies the detected macro-porosity was always larger than micro-defects [22,23], so this is a real analysis of the influence of structural porosity and not so much a material analysis. The specimens were manufactured using a high-pressure die-casting (HPDC) process in the CIMOS d.d. factory in Roc (Croatia). The macro-porosity was introduced by varying the process parameters, i.e., by changing the pressure in the final casting phase and by casting the specimens into the cold tool (form).

**Figure 3:**
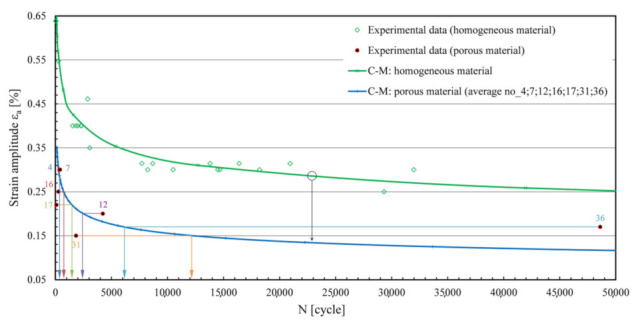
Preliminary assessment of the error in predicting the fatigue life if the strategy is based on a uniform shift of the C–M curve.

The sequence of steps in the implementation of Method B shown in Figure 2 is an interesting and simple way to estimate the fatigue life of porous structures, as it is based on processing the homogeneous composition of the sample under consideration. In this method, various porosity effects are taken into account by a reduced level of the C–M curve of the material. Just like any basic C–M curve of a homogeneous material, the parameters of a uniform lower C–M curve of a porous material can also be estimated with a real-valued genetic algorithm (RVGA [24]); see Figure 3. However, as the pores vary randomly from sample to sample, a larger scatter of measurement points usually results in a worse fatigue life prediction (see Figure 3). In the field of low cyclic fatigue strength (the steepest part of the C–M curve), a good agreement can be expected for samples no. 7 and no. 4, a satisfactory agreement for sample no. 16, while prediction for sample no. 36 is likely over-conservative and non-conservative for sample no. 31, etc. 

**Figure 4:**
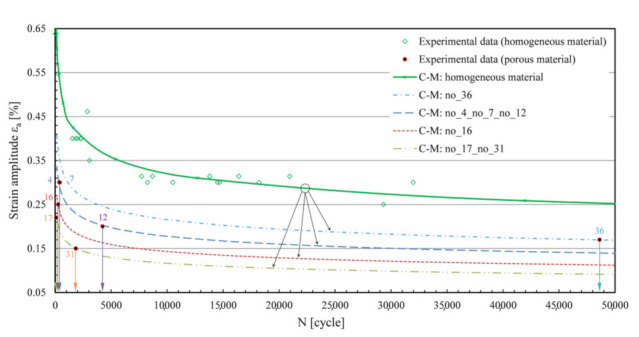
Individual, porosity-adjusted shifts of the C–M curves are a better strategy for predicting the fatigue life.

As can be seen from the preliminary analysis in Figure 3, when using a uniform C–M curve for a porous material, greater discrepancies of results can be expected; hence, the idea that the predictions can be improved if the strategy is modified and adapted for more individual treatment of porous samples. Figure 4 shows the expected values of the lowered levels of the C–M durability curves of AlSi9Cu3 material from the homogeneous to the porous state, formed on the basis of experimental observations [14]. The main challenge in this part is achieving these values analytically as close as possible, i.e., keeping the modelled C–M *ε*_a_–*N* curves as close as possible to the experimental results. 

**Figure 5:**
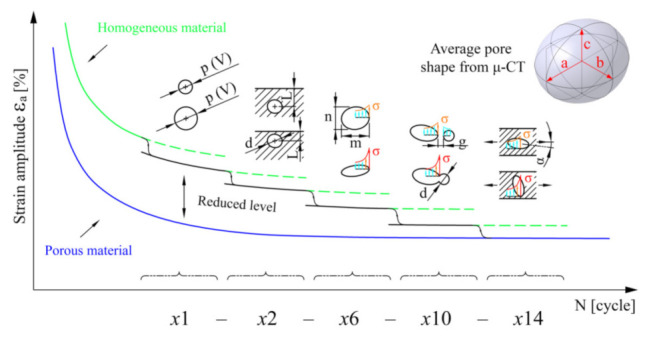
Shift of the Coffin–Manson *ε*_a_–*N* durability curve due to the presence of individual geometric porosity influencing factors.

The correct shift of the C–M curve is crucial, as excessive deviations from the experimental data can lead to larger discrepancies in the prediction of the fatigue life, especially when moving towards the high cycle fatigue (HCF) region, where the C–M curve sections have a lower slope, and this gives a larger Δ region, within which the predicted number of cycles *N* is expected. The final, crucial part is still missing (i.e., a procedure for modelling appropriate synthetic Coffin–Manson curves), so a method is presented in the next chapters, which can be used to make a good estimate of the new adapted curve by taking into account all known influences of porosity. A similar approach that includes a phase for modelling the shift of a synthetic *S*–*N* curve is described in the DNVGL-ST-0361 standard [25]. The adjustment of the durability curve of material on the basis of the ratio between the stress amplitude and the maximum normal stress is described by Susmel [26]. Our methodology for adjusting the level of the C–M curve for porous material is based on taking into account the local geometric characteristics of porosity. The shift is performed on the basis of the size, position, and orientation of the pores, which effects on the yield of the material are based on the results of Murakami and Endo [27] and our research [14]. Five different geometric influences (shown in Figure 5) have been combined, and they have been evaluated at four strain levels *ε*_a_. Individual geometric parameters *x*i are presented in Table 1. 

The theory is derived in the form of equations, by which the appropriate shift of the C–M curve is made. At the same time, numerical simulations of the response are performed for individual geometric porosity parameters and their combinations.

### 2.3. AlSi9Cu3 Alloy

**Figure 6:**
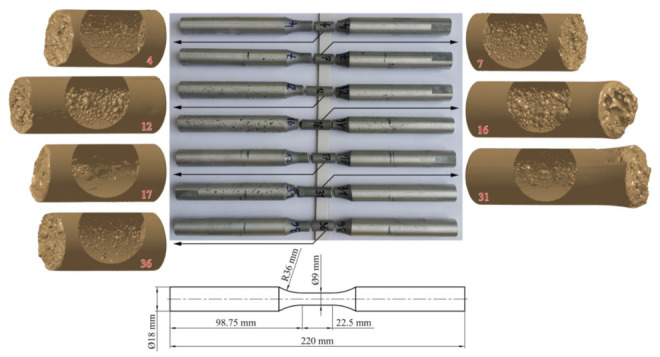
Specimen geometry (**bottom**), LCF-tested specimens (**top**), *μ*-CT-scanned samples for analysis of results (**left**/**right**).

Our tests have been carried out on AlSi9Cu3 alloy, which has a good die-casting ability [28] and is, therefore, widely used in the automotive industry. In a homogeneous state, the alloy has the following static properties [14]: *σ*_y_ = 178 MPa, *σ*_u_ = 322 MPa and *E* = 79,631 MPa. The density of the alloy is *ρ* = 2.76 g/cm^3^, and the Poisson’s ratio *ν* = 0.33. Tests were carried out according to the ASTM E606 standard [29]. The alloy was cast into the standard specimen shape shown in Figure 6. Two series of the specimens were made: (i) quasi-homogeneous specimens, which were used to obtain the durability curve of the base material, and (ii) porous specimens, for which the pores inside were deliberately created by varying the pressure and temperature of the die casting mould, to study the effect of porosity on the dynamic durability. In general, the geometric irregularities present in AlSi9Cu3 alloy are much larger than the material defects, which has a decisive influence on the extent and size of the damage. Macro-porosity is larger than the metallurgical micro-defects, so this method allows a direct assessment of the effect of macro-porosity on the strength of the material. Seven samples analysed in detail with identification numbers 4, 7, 12, 17, 31, and 36 can be seen in Figure 6. The porosity structure inside the samples was determined by the *μ*-CT analysis on a General Electric (GE) V|tome|x s 240. The scanned porosity was then described geometrically by ellipsoids [14] so that the size, position and orientation were known for each pore (i.e., the associated ellipsoid).

### 2.4. Low-Cycle Fatigue Life

All porous specimens in Figure 6 have been tested using the high-strain, low-cycle fatigue (LCF) procedure [14,28] under alternating load (*R* = −1) to failure. The tests were carried out on an MTS servo-hydraulic machine with an extensometer MTS 632.53F-14 installed, with gauge length *l*_0_ = 12 mm [28]. Load amplitudes *ε*_a_ varied from sample to sample, from 0.15 to 0.3%. The values of *ε*_a_ and the experimentally determined numbers of cycles to failure *N*_exp_ are listed in Table 1, and these results can be seen in Figure 3 and Figure 4. Table 1 also shows the geometric characteristics *x*1-*x*2-*x*6-*x*10-*x*16 of the critical pore, which were extracted from the *µ-*CT scans of the porous specimens in Figure 6 using the voxel technique [14] and then modelled with ellipsoids in CATIA V5 software. These characteristics are described in Section 3. If the schematic procedure for the fatigue life estimation of a porous structure in Figure 2 is followed, a cyclic curve for the transformation of the stress *σ* to the strain *ε*_a_ is also needed in an intermediate step. The homogeneous AlSi9Cu3 alloy has Ramberg–Osgood cyclic curve coefficients *K′* = 542 MPa and *n′* = 0.0979 [14,28]. Strain is modelled by equation (Equation (1)) as:

(1) $$\varepsilon =\frac{\sigma }{E}+{\left(\frac{\sigma }{K^{\prime }}\right)}^{\frac{1}{n^{\prime }}}$$

The values of the coefficients of the Coffin–Manson ratio in homogeneous form (Equation (2)) are: *σ′*_f_ = 1087 MPa, *ɛ′*_f_ = 0.0001, *b* = −0.152 and *c* = −1.605 [14,28]. The Coffin–Manson (C–M) curves of the homogeneous samples are shown in Figure 3 and Figure 4.

(2) $$\varepsilon_{a}=\frac{{\sigma^{\prime }}_{f}}{E}{\left(2\cdot N_{f}\right)}^{b}+{\varepsilon^{\prime }}_{f}{\left(2\cdot N_{f}\right)}^{c}$$

After determining all the parameters of the AlSi9Cu3 alloy in homogeneous form, the most important phase follows: the determination of the coefficients of the C–M curve for the porous structure, which is the main topic of the next chapters of this paper.

## 3. Numerical Simulations

A review of the literature shows that most data on dynamic durability are limited to homogeneous structures. For porous-cellular structures, most of the available literature on fatigue life is related to different 2D samples [30,31]. There are some data on the durability of 3D porous structures [32], with analyses of crack development due to different influences [33]. In our paper, we want to address the influence of completely random 3D porosity by numerically modelled *ε*_a_–*N* durability curves. Two types of numerical simulations need to be carried out to do this comprehensively: (i) simulations of the response by individual influences and (ii) simulations of the response of a combination of influences.

### 3.1. Separate Simulations for Each Geometric Parameter

With the use of simulations, the effect of five geometric parameters *x*i on the fatigue life is first assessed separately at different strain values *ε*_a_. The results of these simulations are summarised in Table 2, sorted by levels: in the vertical direction, the individual geometric influences are listed in the ascending order, while in the horizontal direction, the values of the strain *ε*_a_ are listed ascending to the right. The limiting values *ε*_a,1_ and *ε*_a,4_ are taken from the minimum (0.131%) and maximum (0.282%) loading values of the experimental specimens in Table 1. The values *ε*_a,2_ (0.181%) and *ε*_a,3_ (0.231%) are calculated incrementally at regular intervals using Equation (3). The full range of *ε*_a,i_ values is defined as:

*ε*_a,i_ = {*ε*_a,min_; (*ε*_a,min_ + (*ε*_a,max_ − *ε*_a,min_)/3); (*ε*_a,min_ + 2(*ε*_a,max_ − *ε*_a,min_)/3); *ε*_a,max_} (3)

**Figure 7:**
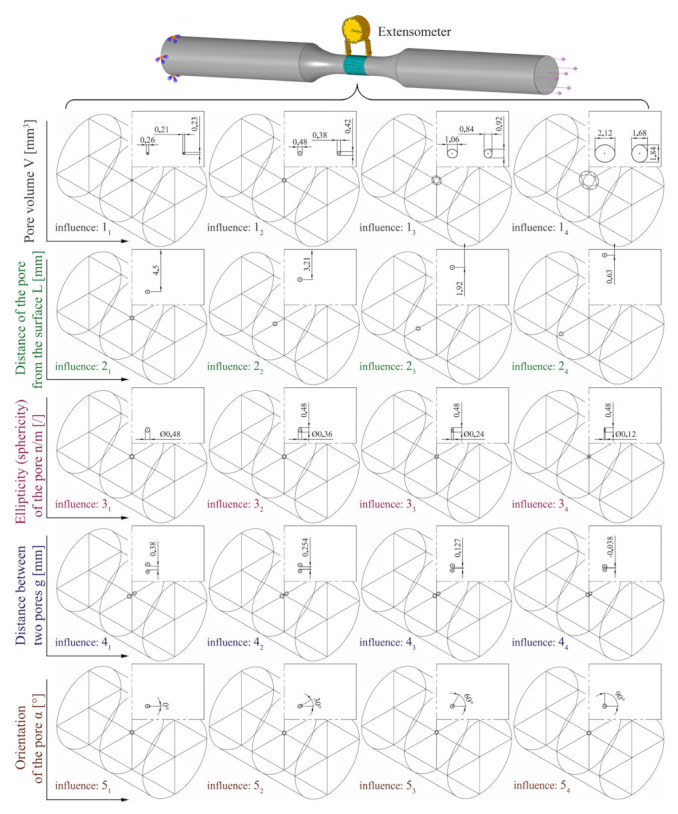
Analysis of different geometric influences of porosity on fatigue life.

In addition to experimental data on *ε*_a_ values, data from numerical simulations are also needed. Figure 7 is helpful here. (*x*1_i_) represents the most important influence: the influence of the pore size (volume) *V* on the fatigue life, etc. There are two drawings in Figure 5 for each influence; the upper case represents a more favourable situation in terms of dynamic durability and the lower a less favourable one. In other cases, the numerical calculations refer to the average pore size on a representative sample no. 12. The half axes of the average pore shape, which can be seen in Figure 5, top right, are: *x* (*a*) = 0.23968 mm, *y* (*b*) = 0.2085 mm, *z* (*c*) = 0.19022 mm. Given the known dimensions of the half axes, the volume of the ellipsoid is calculated as:

*V* = 4/3 · *π* · *a · b · c* (4)

The ratio of volumes of the average pore to the largest pore is as follows for the representative sample no. 12:

*V*_average pore_ ≈ *V*_max_/86 (5)

Likewise, parametric relationships apply between the other geometric dimensions (processed below). As a computational dimension *d* (valid for the examples in Figure 5), the default value of the smallest half axis of the average pore is *d*_z(2)_ = 2 × 0.19022 = 0.38044 mm. The maximum experimental value is *d*_z(max)_ = *d*_z(4)_ = 1.6812 mm. The full set of parametrically calculated ellipsoid diameters *d*_z(1–4)_ is:

*d*_z(i)_ = {(*d*_z(4)_/8); *d*_z(2)_; (*d*_z(4)_/2); *d*_z(4)_} (6)

To obtain the full spectrum of geometric influences of porosity on fatigue life, a series of routine simulations are performed (maximum number = 5 × 4 × 4 = 80 separate simulations). Five separate porosity influences (1–5), shown in Figure 7, with four geometric sizes (1–4), are simulated at four different strain levels in the range (*ε*_a1_–*ε*_a4_), in the following sequence:

(1_i_) Pore volume at the centre (*x*1_i_): *V* (see Figure 5 and Figure 7). The geometry of the ellipsoid always has such an orientation that, according to Figure 5, the *x* (*a*) half-axis coincides with the direction of the load (hence *α* = 0°).

(1_1_) *V* = *V*_max_/491 ≈ 0.007 mm^3^
(1_2_) *V* = *V*_average pore_ ≈ *V*_max_/86 ≈ 0.040 mm^3^
(1_3_) *V* = *V*_max_/8 ≈ 0.430 mm^3^
(1_4_) *V* = *V*_max_ ≈ 3.436 mm^3^

(2_i_) Distance of the pore from the surface (*x*2_i_): *L* (see Figure 5 and Figure 7). The average volume *V* and the shape of the ellipsoid with orientation *α* = 0° according to Figure 5 are always assumed.

(2_1_) *L* ≈ 10.8·*d*_y(2)_ ≈ 4.5 mm (pore in the centre of the specimen)
(2_2_) *L* ≈ 7.7·*d*_y(2)_ ≈ 3.21 mm
(2_3_) *L* ≈ 4.6·*d*_y(2)_ ≈ 1.92 mm
(2_4_) *L* = 3/2·*d*_y(2)_ ≈ 0.63 mm

(3_i_) Ellipticity of the pore (*x*6_i_): *n/m* (see Figure 5 and Figure 7). The starting point of the analyses is set around the average pore size *V*. The ellipticity *n/m* is calculated by varying the ratio of the two smallest half-axes *b* and *c* according to Figure 5. The ellipsoid always has an orientation α = 90°.

(3_1_) *n/m* = 1 (sphere)
(3_2_) *n/m* = 0.75
(3_3_) *n/m* = 0.5
(3_4_) *n/m* = 0.25

(4_i_) Distance between two pores (*x*10_i_): *g* (see Figure 5 and Figure 7). For this part of simulations pore of average size *V* (*α* = 0°) and a sphere with *d* = *d_z*(2)*_* = 0.38044 mm are always compared to each other at the centre of the sample, i.e., the central axis of the specimen is always in the middle between the two pores with the distance between the pore and the central axis equal to *g*/2.

(4_1_) *g* = *d*_z(2)_ = 0.380 mm
(4_2_) *g* = 2/3·*d*_z(2)_ ≈ 0.254 mm
(4_3_) *g* = 1/3·*d*_z(2)_ ≈ 0.127 mm
(4_4_) *g* = −0.1·*d*_z(2)_ ≈ −0.038 mm (overlapping of pores)

(5_i_) Orientation of the average pore (*x*14_i_): *α* (see Figure 5 and Figure 7). In all cases, this is the average pore size *V* located in the centre of the sample. The angle α is the angle between the central axis of the specimen and the biggest half-axis of the ellipsoid.

(5_1_) *α* = 0°
(5_2_) *α* = 30°
(5_3_) *α* = 60°
(5_4_) *α* = 90°

To estimate the stress-strain distribution in the porous mid-part of the specimens (see Figure 7 above) an implicit finite-element analysis was carried out in Abaqus software. The smallest edge of the finite elements was 0.2 mm long, which enabled satisfactory adaptation of the finite-element mesh to the smallest modelled pores. C3D4 tetrahedron finite elements were used in a combination with the free-meshing technique. For the surfaces of the ellipsoid voids manually applied seeds were used. The global finite element size was selected in such a manner that each of the 80 + 64 finite element models from Table 1 and Table 2 consisted of approximately 90,000 finite elements and 16,000 nodes. The Besseling multilinear plasticity material model was applied with the plastic flow curve determined on the basis of the cyclic *σ*–*ε* curve for the AlSi9Cu3 alloy from Section 2.3 that was transformed into the true stress/true strain form [14].

**Figure 8:**
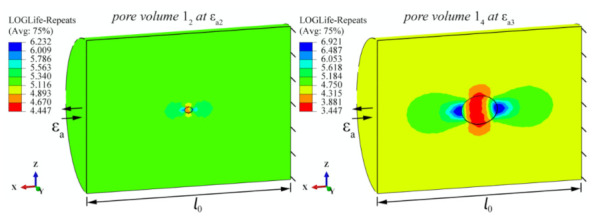
Two examples of numerical calculations of fatigue lives (in LOGLife form) for pores 1_2_ (at *ε*_a2_) and 1_4_ (at *ε*_a3_).

Figure 8 shows two simulated examples of fatigue life calculations at different pore sizes, while Table 2 summarises all 80 results of the numerical simulation series. The proven Brown–Miller (Morrow) criterion [14,34] has been used for the calculation of the fatigue life. The selected fatigue-life damage parameter considers normal strains (Δ*ε*_n_) as well as shear strains (Δ*γ*) in the critical plain, as follows:

(7) $$\frac{∆\gamma }{2}+\frac{∆\varepsilon_{n}}{2}=1.65\frac{\left({\sigma^{\prime }}_{f}-\sigma_{m}\right)}{E}{\left(2\cdot N_{f}\right)}^{b}+1.75\cdot {\varepsilon^{\prime }}_{f}{\left(2\cdot N_{f}\right)}^{c}$$

An important factor that influences the crack initiation is also surface roughness. The surface finish factor *κ*_t_, which depends on the tensile strength *σ*_u_ and surface roughness *R*_a_, a value of *κ*_t_ = 1.02 was selected [35]. The same roughness factor was presumed for the outer specimen surface as well as internal surfaces of the pores. The factor *κ*_t_ is defined in the SIMULIA fe-safe software, which was used for calculating the fatigue damage *D*. The damage calculations *D* are converted to the achieved number of cycles *N*, and the maximum stress concentrations in MPa are also given. An estimate for the shift of the Coffin–Manson curve is then made on the basis of the numerical simulations of the number of cycles reached. For example, when estimating the fatigue life *N* based on the effect of the pore size *V*, an array of 1_1–4_ × *ε*_a1–4_ results is obtained (Table 2). From the array (database) of tabular results, the corresponding number of cycles *N* can be predicted on the basis of the detected pore volume *V*_i_ and the strain *ε*_i_ of the analysed sample.

**Figure 9:**
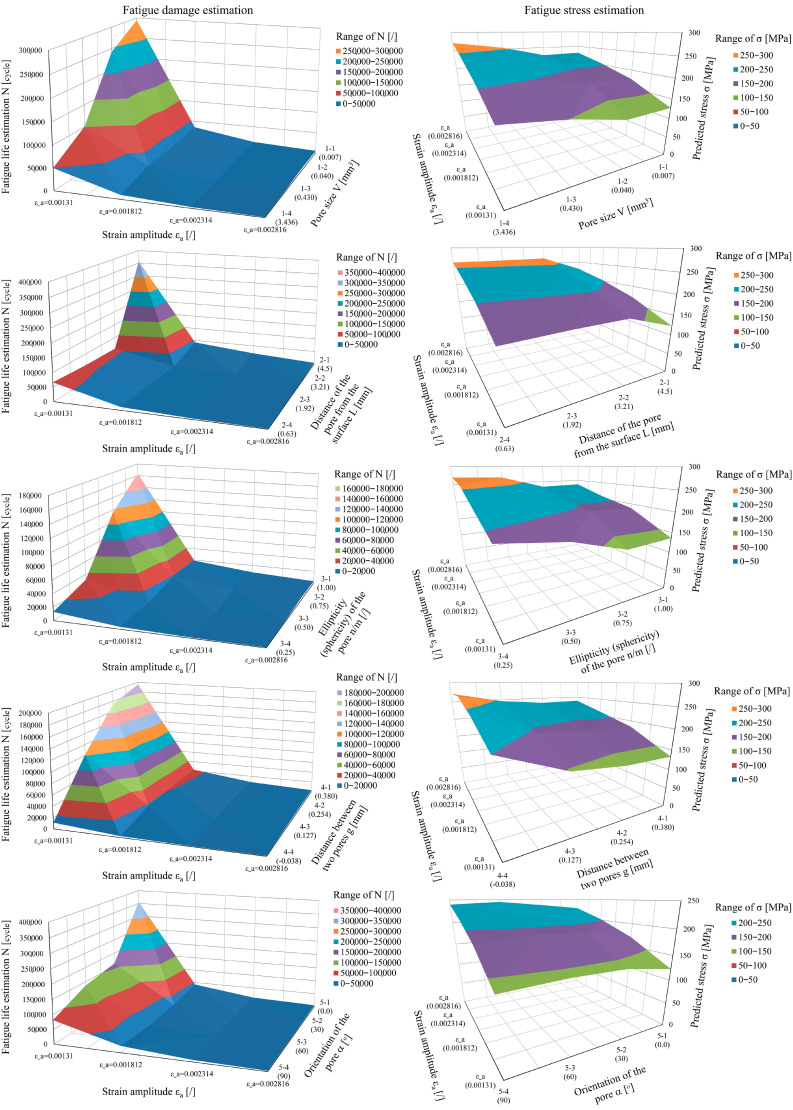
Distribution of fatigue lives *N* (**left**) and stress concentrations *σ* (**right**) as a function of the geometric influences of porosity and strain *ε*_a_.

In addition to the damage calculation (i.e., the number of cycles reached *N*), Table 2 lists the estimated stress peaks *σ*. The graphical analysis in Figure 9 shows the spatial linear interpolation of the points in Table 2; it can be seen that there are significant discrepancies in the shapes of the response surfaces representing the C–M curves for different levels of the individual geometrical characteristics of the critical pores. The distribution of the stresses *σ* around the pores indicates continuous changes of values, whereas these changes are much more variable and pronounced when analysing the final *N* cycles. A comprehensive analysis of the experimental and theoretical data suggests that the methodology for estimating the fatigue life should be designed on the basis of numerical analyses of the damage *D* or calculations of the cycles *N* (where *N* = 1/*D*) and not just on the basis of a general estimate of the stress *σ*.

From the examples in Figure 9 left it can be seen that, as the strain *ε*_a_ increases, a distinct limit is reached, where we can see a transition from the steeper to the flatter part of the surfaces, representing the number of cycles reached *N*. This partly coincides with the transition of the material from the elasto-plastic to the elastic region. The transition is more pronounced for smaller pores, where proportionally, due to the smaller pore volume, there are also smaller proportions of material plastification zones, while a higher proportion of material plastification occurs already at lower strain (lower *ε*_a_ values) for larger pore volumes.

### 3.2. Simulations of Influences of Parameter Combinations on the Fatigue Life

In the research conducted thus far, we have defined five main geometric parameters (and a sixth one: the strain *ε*_a_) that have the greatest impact on the lowered level of the C–M curve of a porous material. Next, we attempt to take a step forward and combine the influences of different factors and determine the overall measurable effect that will be reflected in the correspondingly lower level of the C–M curve of the material, from the initial state of homogeneity to the corresponding state of porosity (see Figure 4).

After analysing the separate effects of the individual geometrical characteristics of the pores on the fatigue life, these partial effects are combined into a single synthetic Coffin–Manson porosity durability curve for a given specimen. Taguchi orthogonal arrays are used for this purpose. One of the advantages of the Taguchi method [36] is the introduction of orthogonal arrays [37,38], which can considerably reduce the required number of tests without losing information. Orthogonal arrays *L*_n_ (*Y^x^*) of different shapes are used. The notations mean: n—number of simulations needed, where up to *X* porosity influential parameters at *Y* levels are varied. In our case, the orthogonal array *L*_64_ (4^21^) was used. With this analysis, a total of 5 + 1 factors or influences are checked. The five factors represent geometric parameters *f*1–5, with an additional sixth factor (+1) influencing *f*6, representing the varying strain *ε*_a_. The jointly formed chain of combined influences by lines has the form:

*f*1(1–4)-*f*2(1–4)-*f*3(1–4)-*f*4(1–4)-*f*5(1–4)-*f*6(1–4) (8)

which is equivalent to the original Taguchi array *L*_64_ (4^21^) in Table 3:

*x*1_(1__–__4)_-*x*2_(1__–__4)_-*x*6_(1__–__4)_-*x*10_(1__–__4)_-*x*14_(1__–__4)_-*x*18_(1__–__4)_ (9)

**Figure 10:**
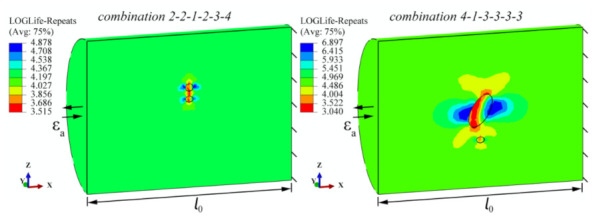
Two examples of numerical calculations of fatigue lives (in LOGLife form) for the combinations of influences 2-2-1-2-3-4 and 4-1-3-3-3-3-3.

**Figure 11:**
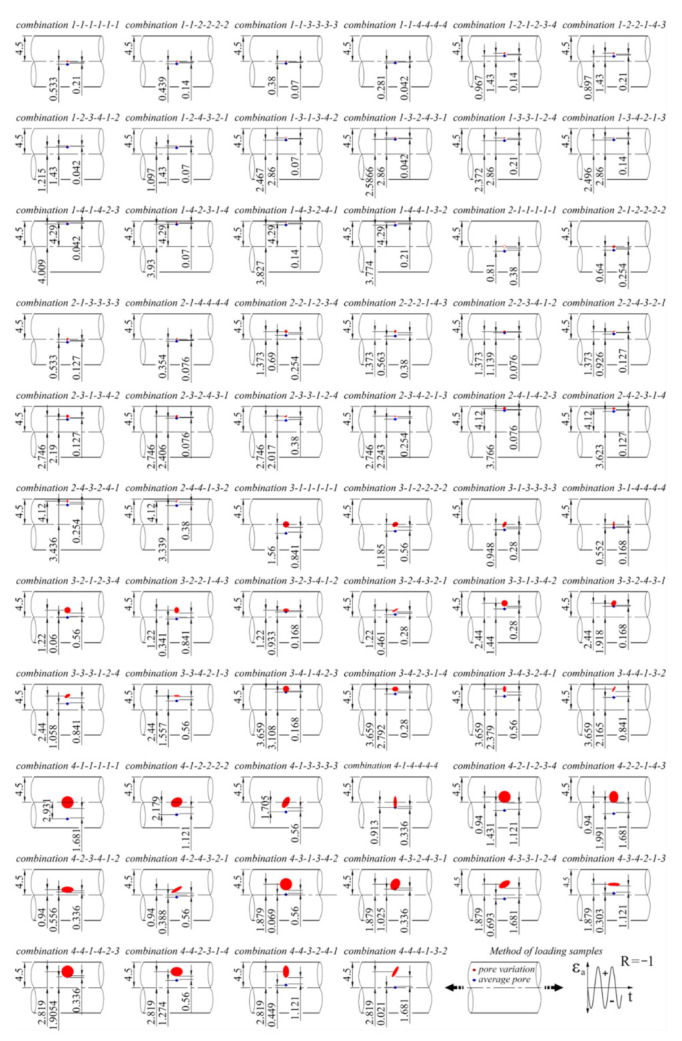
The full range of simulated pore combinations for the Taguchi method.

The factor *x*i represents the column of the Taguchi array *L*_64_ (4^21^), and the index (1–4) represents the range of quantities that vary within each influence. These six factors were defined at four levels or sizes, as shown in the previous Section 3.1. In this orthogonal array set up, which can be seen in Table 3, the combinations run from 1-1-1-1-1-1 to 4-4-4-1-3-2. In practice, this means that there are always two pores involved in a single simulation of a given combination, which are positioned relative to each other in size, shape and orientation according to a given arrangement. One of the pores always has the average shape and size of the *V*_average pore_. Two examples of the fatigue life calculations of a combination of pores from an orthogonal table can be seen in Figure 10. The fatigue life was calculated in the same way as in Section 3.1. A presentation of all combinations of simulated pore shapes is shown in Figure 11. 

### 3.3. Formulation of Equations to Calculate the Shift of the Synthetic ε_a_–N Curve due to Porosity Effects

Using a large database of 144 simulations (80 simulations for the basic parameters and 64 simulations based on the Taguchi orthogonal array), the variation of the Coffin–Manson (C–M) curve as a function of the geometric parameters can be made in such a way to fit the simulated data in the best manner. The first finding is that the inverse value of the pore-to-surface distance (*L*→1/*L*; i.e., parameter *x*2 in the Taguchi array) and the inverse value of the distance between two pores (*g*→1/*g*; i.e., the parameter *x*10 in the Taguchi array) must be taken into account in the approximation model. In this case, the modelling of the influence of the pore geometry parameters on the position of the C–M curve is significantly improved.

As the notch effect coefficient decreases from high-cycle fatigue strength to low-cycle fatigue strength and is practically equal to 1 under static load or at ultra-low cycle fatigue strength [39,40], this means that there is a transition of the curve slope in the LCF area. Therefore, both the exponent *b* of the elastic term and the parameter *σ′*_f_ must be varied in the Coffin–Manson equation (Equation (2)). The parameters of the plastic term of the equation (*ε′*_f_ and exponent *c*) are retained; thus, a reduction in the notch effect factor in the region of low-cycle fatigue strength is achieved as well as the increased sensitivity of the material to the notched geometry in the high-cycle domain. Such fatigue behaviour is typical for less ductile materials and it can be observed from experimental results that the AlSi9Cu3 alloy is relatively brittle. The parameters of the elastic term of the equation are changed as a function of the porosity parameters, which were as follows in our case (summarised from Figure 5):

(1) Pore size *V* (parameter *x*1 in the Taguchi array);
(2) Distance of the pore from the surface of the specimen *L* (parameter *x*2 in the Taguchi array);
(3) Pore ellipticity *n/m* (parameter *x*6 in the Taguchi array);
(4) Distance between two pores *g* (parameter *x*10 in the Taguchi array);
(5) Orientation of the pore *α* (parameter *x*14 in the Taguchi array).

To determine the functional dependence of the parameters *σ′*_f_ and *b* of the Coffin–Manson equation, it is necessary to transform the C–M equation (Equation (2)) into the following form:

(10) $$\varepsilon_{a}-{\varepsilon^{\prime }}_{f}\cdot {\left(2\cdot N_{f}\right)}^{c}=\frac{{\sigma^{\prime }}_{f}}{E}\cdot {\left(2\cdot N_{f}\right)}^{b}$$

(11) $$\underset{K\left(N_{f}\right)}{\underbrace{\left[\varepsilon_{a}-{\varepsilon^{\prime }}_{f}\cdot {\left(2\cdot N_{f}\right)}^{c}\right]}}\cdot E={\sigma^{\prime }}_{f}\cdot {\left(2\cdot N_{f}\right)}^{b}$$

(12) $$Y=log_{10}\left[K\left(N_{f}\right)\cdot E\right]=log_{10}{\sigma^{\prime }}_{f}+b\cdot log_{10}\left(2\cdot N_{f}\right)$$

Given the structure of equation (Equation (12)), it is possible to deal with the effects of the above-mentioned five porosity parameters on the variation of the *σ′*_f_ and *b* parameters of the Coffin–Manson equation independently by two multivariate linear regression models:

(13) $$log_{10}{\sigma^{\prime }}_{f}=s_{0}+s_{1}\cdot x1+s_{2}\cdot \left(\frac{1}{x2}\right)+s_{3}\cdot x6+s_{4}\cdot \left(\frac{1}{x10}\right)+s_{5}\cdot x14$$

(14) $$b=b_{0}+b_{1}\cdot x1+b_{2}\cdot \left(\frac{1}{x2}\right)+b_{3}\cdot x6+b_{4}\cdot \left(\frac{1}{x10}\right)+b_{5}\cdot x14$$

*s*_i_; i = 0, …, 5 are the regression coefficients for calculating the parameter *σ′*_f_, *b*_j_; j = 0,…,5 are the regression coefficients for calculating the parameter *b*. The estimation of the regression coefficients in equations (Equations (13) and (14)) is carried out on the basis of 144 simulation results in the form of the following dataset:

(15) $$\left\{{\left(x1,x2,x6,x10,x14,N_{f}\right)}_{k};k=1,\ldots ,144\right\}$$

Based on the combined model in the equation below:

(16) $$Y=log_{10}\left[K\left(N_{f}\right)\cdot E\right]=log_{10}{\sigma^{\prime }}_{f}+b\cdot log_{10}\left(2\cdot N_{f}\right)=\;=s_{0}+s_{1}\cdot x1+s_{2}\cdot \left(\frac{1}{x2}\right)+s_{3}\cdot x6+s_{4}\cdot \left(\frac{1}{x10}\right)+s_{5}\cdot x14+\;+\left[b_{0}+b_{1}\cdot x1+b_{2}\cdot \left(\frac{1}{x2}\right)+b_{3}\cdot x6+b_{4}\cdot \left(\frac{1}{x10}\right)+b_{5}\cdot x14\right]\cdot log_{10}\left(2\cdot N_{f}\right)$$

the regression parameters were assessed using the IBM SPSS software. The target function was the sum of the squares of distances between the simulated and regression-modelled results. For our specific case of 144 simulations, equations (Equations (13) and (14)) have the following forms:

(17) $$log_{10}{\sigma^{\prime }}_{f}=2.771+0.025\cdot x1-0.014\cdot \left(\frac{1}{x2}\right)+0.200\cdot x6+0.006\cdot \left(\frac{1}{x10}\right)-0.020\cdot x14$$

(18) $$b=-0.137-0.015\cdot x1-0.003\cdot \left(\frac{1}{x2}\right)-0.029\cdot x6-0.003\cdot \left(\frac{1}{x10}\right)-0.002\cdot x14$$

If equations (Equations (17) and (18)) are inserted into equation (Equation (12)), the correlation coefficient of the model based on 144 simulations is equal to *R* = 0.8991, and the corresponding determination coefficient is equal to *R*^2^ = 0.8092. This means that the regression models for the parameters *σ′*_f_ and *b* of the Coffin–Manson equation describe more than 80% of the total variance of the simulated results for the prediction of the fatigue life if macro-pores are present. If the pairwise interactions from the Taguchi *L*_64_ (4^21^) array were also included in the regression models of Equations (13) and (14) the quality of the combined model of Equation (16) would be only marginally improved (*R* = 0.9132; *R*^2^ = 0.8341). However, such an improvement does not justify extending the model with additional pairwise interactions, because they do not significantly correct the calculation of the *σ′*_f_ and *b* parameters.

## 4. Comparison of Theoretical and Experimental Results

The acquired porosity data were, due to the combined action of different pore notch effects, first simulated with FEM and then converted to modified Coffin–Manson coefficients, based on regression calculations, using Taguchi combinations. An additional advantage of the presented method is that it can also be applied in cases of isolated single pores or other local anomalies, such as inclusions. One of the most important objectives of the study was to determine the influence of the accuracy of different geometrical porosity factors on fatigue life. The most important criterion in the modification of the Coffin–Manson (C–M) durability curve *ε*_a_–*N* is the shape of the pores. The research and the results of the dynamic durability *N* in Figure 9 show, for values around *n/m* ≈ 0.5, a clear characteristic division into two main influential groups of porosity: the more favourable spherical pores, and in contrast, disc-shaped (flat) pores, which produce much more significant local plastification, especially if the longest axis of the ellipsoid is not aligned with the loading direction.

At the critical points, a strong increase in notch effects can be observed from the stress analysis, leading to a rapid fatigue failure. It can also be seen from Figure 9 that even a small off-centre position of the pores in the specimen results in a large increase of damage *D*. Similarly, even a small rotation of the pore away from the neutral axis (α > 30°) results in a noticeable decrease in fatigue life. Based on the results, we believe that the presented method is more suitable for dealing with gas porosity, where more regular pore shapes are present. Shrinkage porosity tends to be more diffuse and irregularly shaped, making it more difficult to predict the behaviour of the material and to lower the level of the C–M curve properly. Moreover, asymmetrically placed pores in the structure, even if volumetric (sphere-like), can be more dangerous, as is the case in sample no. 16, in which the pores are larger at one half of the critical cross-section *A* than at the other. The presence of similarly sized pores of similar volume, even if the degree of porosity is greater, is a more favourable condition due to the more symmetrical, uniform elongations of the structure. 

**Figure 12:**
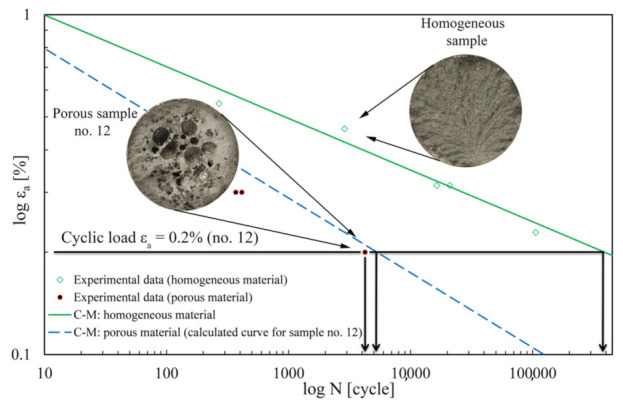
Shift of the calculated C–M curve of AlSi9Cu3 alloy for porous sample no. 12.

**Figure 13:**
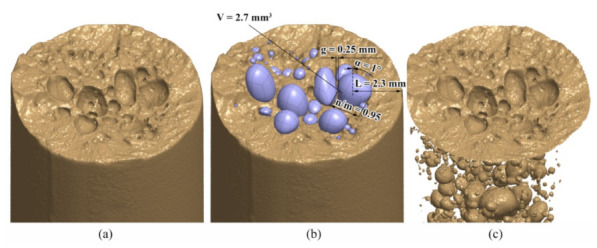
Assessment of critical areas with dimensions around the largest pore in sample no. 12. (**a**) raw data, (**b**) reconstruction of pores on the critical plane, (**c**) examination of the pores inside.

Figure 12 shows an example (as described in Section 3.3) of the shift of the AlSi9Cu3 C–M durability curve for the representative porous sample no. 12. The shift is performed by calculating the terms *σ′*_f_ and *b* using equations (Equations (13) and (14)), into which the regression coefficients *s*_i_, *b*_j_ are inserted, calculated for the general porosity state of the AlSi9Cu3 alloy. Based on this, equations (Equations (17) and (18)) are derived, and then the locally observed peculiarities of the critical pore values, as seen in Figure 13, e.g., for sample no. 12, are inserted, which further improves the trend (shape) of the durability curve. The newly modelled lowered level of the synthetic C–M curve for sample no. 12 now has values *σ′*_f_ = 1063 MPa, *b* = −0.22, while the other two parameters of the C–M equation are retained: *ε′*_f_ = 0.0001, *c* = −1.605. In some cases, such as sample no. 4, with a dominant large pore in the centre, there is no doubt that a potential critical failure location lies around it. If there is any doubt as to where the most critical part is, several quick calculations can be made using the method presented, taking into account the combination of geometric influences that gives the shortest prediction of the fatigue life.

It can be seen from the results in Figure 12 that the shift of the synthetic C–M curve for the typical porosity of specimen no. 12 is slightly non-conservative. We estimate this is because the shift of the synthetic curve is based only on the interaction of two pores, one of which has an average size. However, since there is an interaction of pores significantly larger than average for specimen no. 12 (see Figure 13), the actual fatigue life is slightly shorter than that predicted by the shift of the C–M durability curve. Porous sample no. 12 has experimentally withstood 4243 cycles, while we estimated about 5000 cycles using the presented method. If we had used dedicated software (e.g., SIMULIA fe-safe, nCode, etc.), which also allows defining the surface condition of the specimen and if we had used one of the specific criteria to deal with failure due to cyclic strain at the critical plane, the predictions of the fatigue-life *N* could have been improved further. As can be further seen from Figure 12, a homogeneous sample withstands around 350,000 cycles at the same load *ε*_a_ = 0.2%. As part of the data comparisons, a test duplicate method was performed, where we compared the influence of two different criteria for predicting the fatigue life of our specimens, i.e., the before-mentioned Brown–Miller–Morrow criterion and the well-known Smith–Watson–Topper criterion (Equation (19)) for the same boundary conditions.

(19) $$\sigma_{\max}\cdot \varepsilon_{a}=\frac{{\left({\sigma^{\prime }}_{f}\right)}^{2}}{E}{\left(2\cdot N_{f}\right)}^{2b}+{\sigma^{\prime }}_{f}\cdot {\varepsilon^{\prime }}_{f}{\left(2\cdot N_{f}\right)}^{b+c}$$

Two combinations of pores (i.e., 2-2-1-2-3-4 and 4-1-3-3-3-3 from Figure 10) were analysed using the two criteria. Based on the data obtained in Table 4, it can be concluded that the results of the calculated fatigue lives *N* are comparable for both methods. The Brown–Miller–Morrow criterion is slightly more conservative, which means that we are on the safer side of predicting the fatigue life when using it. Additional discussion of results for both tested criteria is presented in Table 4.

**Figure 14:**
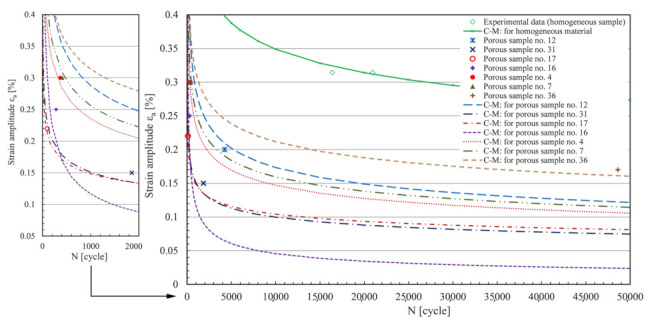
Display of the calculated shift of the C–M curves for all porous samples analysed.

Figure 14 shows the other samples processed in the same way. What was only a prediction in the introduction can now be confirmed: the best match of the experimental points with the modelled synthetic curves is obtained at the highest strain amplitudes *ε*_a_, as is the case for samples no. 4, 7, 16 and 17, where the curve sections are the steepest. A very good result was obtained for sample no. 36, where the smallest pores are present at lower cyclic strains *ε*_a_, where the curve sections are flatter. This indicates the applicability of the method over the whole elasto-plastic range of the material and for pores of different sizes. The largest deviation of the modelled synthetic curve from the corresponding experimental point is observed for sample no. 31. In this specific case, a lamellar structure was present, whose geometry is difficult to describe accurately using ellipsoids, yet the estimated deviation is within the double value of the error of the predicted loading cycles to failure. For all specimens, the calculated values of the coefficients and exponents of the modelled synthetic C–M durability curves are summarised in Table 1.

## 5. Conclusions

From the observations of the experimental testing of the porous structures and the equivalent numerical simulations of the response of the specimen geometry, the main highlights of the study can be summarised in a few points:

- The method presented is an efficient and simple procedure for the estimation of the fatigue life, since the treatment of porosity starts from a homogeneous geometry, where fewer finite elements are used, and the influence of porosity is incorporated by a precise shift of the Coffin–Manson (C–M) *ε*_a_–*N* durability curve.
- As we have seen from experimental data, because of the large differences in the durability of differently porous samples, it is not reasonable to base the fatigue life calculation around one averagely shifted C–M curve because the predictions may be either too conservative or too optimistic. This brings us to the idea of individual treatment of a particular porous case.
- Modelling the synthetic durability C–M curve of a porous material based on regression calculations, its new terms *σ′*_f_ and *b*, represents a significant advance in durability predictions according to the LCF theory since a simple preliminary analysis can give a reasonably good estimate of the fatigue life of a given porous specimen.
- The presented method allows the modelling of a synthetic C–M durability curve on two levels. By regression calculations of the coefficients *s*_i_, *b*_j_, the basic course of the material durability curve is obtained first, which is then individually refined by entering the geometrical parameters *x*1–14 for a given analysed specimen. When studying porosity in AlSi9Cu3 alloy, it can be concluded from the simulations and regression analyses that this material is most sensitive to the geometric parameter of the orientation of the disc-shaped pores, defined by the regression coefficients *s*_5_ and *b*_5_. It is, therefore, important, when examining the porosity, to estimate as best as possible the angle *α* of the orientation of the dominant pores within the geometric influence *x*14.
- The prediction of the fatigue life is generally good on all samples analysed. The result is slightly more conservative only for sample no. 31, where the effect of the lamellarity of the structure around the central axis is more difficult to describe analytically via the ellipsoids used.

## Figures and Tables

**Table 1:** Geometric characteristics of the critical pores and the fatigue life of the tested samples.

Sp. No.	Strain *ε*_a_ [%]	Number of Cycles Reached *N*_exp_	Volume of the Critical Pore ^‡^ *V* [mm^3^]	Distance of the Pore from the Surface *L* [mm]	Ellipticity of the Pore*n/m* [/]	Distance between Two Pores *g* [mm]	Orientation of the Pore *α* [°]	C–M of the Porous Structure	
			*x*1	*x*2	*x*6	*x*10	*x*14	*σ′*_f_	*b*
12	0.2 * (0.19 **)	4243	2.7	2.3	0.95	0.25	1	1063	−0.22
31	0.15 * (0.13 **)	1853	0.17	4.5	0.35	0.5	12	411	−0.18
36	0.17 * (0.16 **)	48,619	0.11	2.2	0.85	1	2	801	−0.172
17	0.22 * (0.21 **)	98	1.3	4	0.1	–0.1	12	331	−0.154
16	0.25 * (0.25 **)	282	16.7	2.5	0.9	–0.2	4	1790	−0.408
4	0.3 * (0.28 **)	370	0.83	3.6	0.95	0.2	6	772	−0.205
7	0.3 * (0.28 **)	415	1.3	3.8	0.9	0.2	4	851	−0.206

* Theoretically determined value. ** The actual average experimental values obtained on the MTS extensometer. **^‡^** The biggest pore, which was detected by *μ*-CT scanner was considered as a critical pore.

**Table 2:** Separate prediction of the reached stress *σ* and the fatigue life *N* for each geometric influence and strain levels *ε*_a_.

(1) Pore Size (Volume) *V*								
/	(6) Strain Level *ε*_a_							
	*ε*_a1_ = 0.00131		*ε*_a2_ = 0.001812		*ε*_a3_ = 0.002314		*ε*_a4_ = 0.002816	
	Stress *σ* [MPa]	Cycles *N* [Cycle]	Stress *σ* [MPa]	Cycles *N* [Cycle]	Stress *σ* [MPa]	Cycles *N* [Cycle]	Stress *σ* [MPa]	Cycles *N* [Cycle]
1_1_	127	5.468 * (293,764)	175	4.536 * (34,356)	204	4.061 * (11,508)	227	3.689 * (4887)
1_2_	122	5.384 * (242,103)	181	4.447 * (27,990)	209	3.966 * (9247)	225	3.712 * (5152)
1_3_	149	5.004 * (100,925)	197	4.433 * (27,102)	226	3.689 * (4887)	254	3.261 * (1824)
1_4_	165	4.702 * (50,350)	208	3.987 * (9705)	241	3.447 * (2799)	271	2.996 * (991)
**(2) Distance of the pore from the surface *L***								
2_1_	123	5.552 * (356,451)	170	4.621 * (41,783)	202	4.294 * (19,679)	226	3.698 * (4989)
2_2_	162	4.751 * (56,364)	206	4.012 * (10,280)	239	3.486 * (3062)	266	3.075 * (1189)
2_3_	161	4.771 * (59,020)	204	4.039 * (10,940)	236	3.506 * (3206)	263	3.082 * (1208)
2_4_	157	4.825 * (66,834)	202	4.053 * (11,298)	233	3.548 * (3532)	261	3.125 * (1334)
**(3) Ellipticity of the pore *n/m***								
3_1_	136	5.214 * (163,682)	188	4.275 * (18,836)	209	3.911 * (8147)	236	3.495 * (3126)
3_2_	131	5.064 * (115,878)	192	4.276 * (18,880)	209	3.953 * (8974)	233	3.577 * (3776)
3_3_	171	4.502 * (31,769)	210	3.845 * (6998)	242	3.331 * (2143)	267	2.973 * (940)
3_4_	190	4.120 * (13,183)	223	3.535 * (3428)	256	3.070 * (1175)	270	2.842 * (695)
**(4) Distance between two pores *g***								
4_1_	133	5.291 * (195,434)	184	4.356 * (22,699)	211	3.888 * (7727)	237	3.473 * (2972)
4_2_	134	5.241 * (174,181)	185	4.323 * (21,038)	209	3.917 * (8260)	235	3.501 * (3170)
4_3_	147	5.005 * (101,158)	193	4.245 * (17,579)	219	3.791 * (6180)	246	3.373 * (2360)
4_4_	200	4.054 * (11,324)	241	3.402 * (2523)	271	2.953 * (897)	271	2.842 * (695)
**(5) Orientation of the pore *α***								
5_1_	123	5.552 * (356,451)	170	4.621 * (41,783)	202	4.072 * (11,803)	226	3.698 * (4989)
5_2_	139	5.207 * (161,065)	190	4.406 * (25,468)	214	3.945 * (8810)	236	3.560 * (3631)
5_3_	141	5.136 * (136,773)	191	4.247 * (17,660)	216	3.820 * (6607)	245	3.374 * (2366)
5_4_	137	4.911 * (81,470)	184	4.356 * (22,699)	215	3.826 * (6699)	240	3.431 * (2698)

* *N* in LOGLife form (Brown–Miller–Morrow criterion used)—e.g., LOGLife of 100 cycles = 2. ( ) number of cycles *N.*

**Table 3:** Prediction of stress magnitude *σ* and fatigue lives *N* for different pore combinations.

Pore Volume *V*	Taguchi Combinations						Stress *σ* [MPa]	Cycles *N* [Cycle]	Pore Volume *V*	Taguchi Combination						Stress *σ* [MPa]	Cycles *N* [Cycle]
	*x*_1_	*x*_2_	*x*_6_	*x*_10_	*x*_14_	*x*_18_				*x*_1_	*x*_2_	*x*_6_	*x*_10_	*x*_14_	*x*_18_		
1_1_	1-1-1-1-1-1						124	5.113 * (129,718)	1_3_	3-1-1-1-1-1						147	5.000 * (100,000)
	1-1-2-2-2-2						188	4.302 * (20,045)		3-1-2-2-2-2						206	3.986 * (9683)
	1-1-3-3-3-3						268	3.001 * (1002)		3-1-3-3-3-3						252	3.192 * (1556)
	1-1-4-4-4-4						271	2.713 * (516)		3-1-4-4-4-4						271	2.555 * (359)
	1-2-1-2-3-4						239	3.479 * (3013)		3-2-1-2-3-4						258	3.187 * (1538)
	1-2-2-1-4-3						208	3.958 * (908)		3-2-2-1-4-3						237	3.487 * (3069)
	1-2-3-4-1-2						217	3.432 * (2704)		3-2-3-4-1-2						214	3.883 * (7638)
	1-2-4-3-2-1						178	4.367 * (23,281)		3-2-4-3-2-1						188	4.047 * (11,143)
	1-3-1-3-4-2						203	4.074 * (11,858)		3-3-1-3-4-2						205	4.033 * (10,789)
	1-3-2-4-3-1						204	3.779 * (6012)		3-3-2-4-3-1						188	3.996 * (9908)
	1-3-3-1-2-4						254	3.252 * (1786)		3-3-3-1-2-4						260	3.161 * (1449)
	1-3-4-2-1-3						207	4.010 * (10,233)		3-3-4-2-1-3						206	4.032 * (10,765)
	1-4-1-4-2-3						252	3.214 * (1637)		3-4-1-4-2-3						259	3.173 * (1489)
	1-4-2-3-1-4						258	3.210 * (1622)		3-4-2-3-1-4						270	3.000 * (1000)
	1-4-3-2-4-1						186	4.288 * (19,409)		3-4-3-2-4-1						201	4.000 * (10,000)
	1-4-4-1-3-2						240	3.342 * (2198)		3-4-4-1-3-2						265	2.928 * (847)
1_2_	2-1-1-1-1-1						128	5.269 * (185,780)	1_4_	4-1-1-1-1-1						181	4.422 * (26,424)
	2-1-2-2-2-2						189	4.269 * (18,578)		4-1-2-2-2-2						217	3.823 * (6653)
	2-1-3-3-3-3						240	3.395 * (2483)		4-1-3-3-3-3						264	3.040 * (1096)
	2-1-4-4-4-4						271	2.812 * (649)		4-1-4-4-4-4						271	2.558 * (361)
	2-2-1-2-3-4						236	3.515 * (3273)		4-2-1-2-3-4						271	2.926 * (843)
	2-2-2-1-4-3						209	3.942 * (8750)		4-2-2-1-4-3						270	2.959 * (910)
	2-2-3-4-1-2						204	4.034 * (10,814)		4-2-3-4-1-2						203	4.052 * (11,272)
	2-2-4-3-2-1						127	5.215 * (16,406)		4-2-4-3-2-1						194	3.896 * (7870)
	2-3-1-3-4-2						192	4.272 * (18,707)		4-3-1-3-4-2						219	3.775 * (5957)
	2-3-2-4-3-1						167	3.741 * (5508)		4-3-2-4-3-1						187	4.318 * (20,800)
	2-3-3-1-2-4						250	3.301 * (2000)		4-3-3-1-2-4						270	2.996 * (991)
	2-3-4-2-1-3						210	3.926 * (8433)		4-3-4-2-1-3						208	3.985 * (9661)
	2-4-1-4-2-3						264	2.990 * (977)		4-4-1-4-2-3						266	3.079 * (1199)
	2-4-2-3-1-4						252	3.306 * (2023)		4-4-2-3-1-4						271	2.978 * (951)
	2-4-3-2-4-1						175	4.489 * (30,832)		4-4-3-2-4-1						192	4.024 * (10,568)
	2-4-4-1-3-2						243	3.308 * (2032)		4-4-4-1-3-2						271	2.796 * (625)

* *N* in LOGLife form (Brown–Miller–Morrow criterion used)—e.g., LOGLife of 100 cycles = 2. ( ) number of cycles *N.*

**Table 4:** A duplicate response for two pore combinations using two different algorithms.

Combinations from Taguchi Array	Brown–Miller–Morrow (B–M–M)	Smith–Watson–Topper (S–W–T)	Comment of the Results
2-2-1-2-3-4	3.515 * (3273)	3.583 * (3828)	When considering pores of the same size with mutual distances *g* < 0.38 mm, the B–M–M criterion gives slightly more conservative results. The reason may be the additional inclusion of the shear stress in the B–M–M criterion, which has an additional impact on the interaction between the proximal pores.
4-1-3-3-3-3	3.040 * (1096)	3.086 * (1219)	If there is a combination of the dominant pores in the middle of the sample and the distance between the pores *g* > 0.38 mm, the criteria B–M–M and S–W–T are comparable, with minimal differences in the prediction of the duration. In this case too, the B–M–M criterion is somewhat more conservative.

* *N* in LOGLife form. ( ) number of cycles *N*.

## Data Availability

The data presented in this study are available on request from the corresponding author.

## Nomenclature

*a,b,c*	[mm]	Half-axes of ellipsoid
*b*	[-]	Fatigue strength exponent
*b*_0,1,2,3,4,5_	[-]	Regression coefficients for estimating the Coffin–Manson exponent *b*
*c*	[-]	Fatigue ductility exponent
*d*	[mm]	Nominal diameter of the smallest half-axle of the ellipsoid
*n/m*	[-]	Sphericity ratio: quotient between the biggest and the smallest axle of the ellipsoid
*n′*	[-]	Cyclic strain hardening exponent
*p*	[-]	Porosity
*s*_0,1,2,3,4,5_	[-]	Regression coefficients for estimating the Coffin–Manson parameter *σ*′_f_
*g*	[mm]	Distance between two pores
*l*_0_	[mm]	Gauge length (extensometer)
*A*	[mm^2^]	Cross-section
*C–M*	[-]	Coffin–Manson curve (equation)
*D*	[-]	Damage variable
*E*	[MPa]	Young’s modulus
*K′*	[MPa]	Cyclic strain hardening coefficient
*L*	[mm]	The smallest distance between a pore boundary and a surface of the structure
*L*_n_	[-]	Taguchi orthogonal array
*N*	[cycle]	Predicted fatigue life (cycles)
*N*_exp_	[cycle]	Observed experimental fatigue life (cycles)
*N*_f_	[cycle]	Number of cycles to failure
*R*_a_	[μm]	Surface roughness
*V*	[mm^3^]	Pore volume
*ε*	[-]	Strain
*ε*_a_	[-]	Strain amplitude
*ε′*_f_	[-]	Fatigue ductility coefficient
*α*	[°]	Pore direction: angle between the specimen longitudinal axis and the direction of the biggest pore half-axle
*ν*	[-]	Poisson’s ratio
*ρ*	[g/cm^3^]	Density
*σ*	[MPa]	Stress
*σ′*_f_	[MPa]	Fatigue strength coefficient
*σ*_m_	[MPa]	Mean stress in the cycle
*σ*_max_	[MPa]	Maximum stress in the cycle
*σ*_u_	[MPa]	Ultimate strength
*σ*_y_	[MPa]	Yield strength
*κ*_t_	[-]	Surface finish factor
*Δl*	[mm]	Length change
*Δε*_n_	[-]	Normal strain
*Δγ*	[-]	Shear strain
